# Evaluation of the rSP03B sero-strip, a newly proposed rapid test for canine exposure to *Phlebotomus perniciosus*, vector of *Leishmania infantum*

**DOI:** 10.1371/journal.pntd.0006607

**Published:** 2018-08-02

**Authors:** Laura Willen, Pascal Mertens, Petr Volf

**Affiliations:** 1 Department of Parasitology, Faculty of Science, Charles University, Prague, Czech Republic; 2 Coris BioConcept, Crealys Park, Gembloux, Belgium; National Institutes of Health, UNITED STATES

## Abstract

**Background:**

Canine leishmaniasis (CanL) is a zoonotic disease, caused by *Leishmania infantum* and transmitted by *Phlebotomus perniciosus* in the Mediterranean basin. Previously, an ELISA based on the *P*. *perniciosus* salivary protein SP03B was proposed as a valid tool to screen for canine exposure to sand fly bites across regions endemic for CanL. Although this approach is useful in laboratory settings, a practical tool for immediate application in the field is needed. In this study we propose the rSP03B sero-strip, the first immunochromatographic test (ICT) in the field of vector exposure able to rapidly screen dogs living in endemic areas for the presence of *P*. *perniciosus* and to aid in the evaluation of vector control programs.

**Methodology/Principal findings:**

The ICT was prepared using the bacterially expressed recombinant protein rSP03B as antigen. For test optimization, pre-immune sera from non-bitten laboratory-bred Beagles were used as negative controls. In order to validate the test, sera from laboratory-bred Beagles experimentally exposed to *P*. *perniciosus* bites were used as positive controls. Additionally, all samples were tested by ELISA using whole salivary gland homogenate (SGH) and the rSP03B protein as antigen. An almost perfect degree of agreement was found between the ICT and the SGH-ELISA. Furthermore, the newly proposed rSP03B sero-strip showed a sensitivity of 100% and a specificity of 86.79%.

**Conclusions/Significance:**

We developed a simple and rapid ICT based on the *P*. *perniciosus* rSP03B salivary protein, able to replace the standard ELISA used in previous studies. Our rSP03B sero-strip showed to be highly sensitive and specific in the detection of antibodies (IgG) against *P*. *perniciosus* saliva. In the future, this test can be employed during large-scale epidemiological studies of CanL in the Mediterranean area to evaluate the efficacy of vector control programs.

## Author summary

The sand fly *Phlebotomus perniciosus* is the principle vector of *Leishmania infantum*, causing canine leishmaniasis in the Mediterranean basin. While the sand fly female takes a blood meal, it injects saliva into the host skin, evoking a specific antibody response in the host. The antibody level in the host correlates with the intensity of exposure to sand flies. Previously, the specific antibody response (IgG) against a salivary protein of *P*. *perniciosus*—SP03B—has been proposed as a valid biomarker to estimate dog exposure to *P*. *perniciosus* in the Mediterranean area. Since standard serological methods are impractical and time-consuming in field conditions, we propose the rSP03B sero-strip—a rapid test that can be immediately applied to screen large cohorts of dogs for the presence of anti-*P*. *perniciosus* antibodies. Our test is the first rapid test in the field of vector exposure, it is highly sensitive and specific and shown to be a valid replacement for standard serological assays. In addition, this test could be used as an evaluation tool for vector control programs.

## Introduction

Canine leishmaniasis (CanL) is a widespread zoonotic disease present in several countries in Latin-America, Europe and Asia [1,2]. It is a severe multi-systemic disease of dogs caused by the protozoan parasite *Leishmania infantum*. The disease manifests itself in variable clinical signs, with the majority of dogs experiencing poor body condition, generalized muscular atrophy, lymphadenomegaly and excessive skin scaling (reviewed in [3]). CanL is endemic across the Mediterranean basin [1,2], with seroprevalences varying from region to region depending on ecological aspects [4]. Overall, 2.5 million dogs are estimated to be infected in southern Europe [4]. Since dogs suffering from the disease are extremely difficult to treat, it is not surprising that the high incidence of CanL in southern Europe represents the main cause of deaths amongst dogs in the region [5]. However, recent occurrence of autochtonous cases in Romania, Hungary and northern Italy suggests that the disease is not limited anymore to the Mediterranean region, but confirms its spread to more northern areas (reviewed in [6]). It is noteworthy that less than 50% of infected dogs develop the disease [7]. However, both sick and asymptomatic dogs represent the main reservoir of the parasite and form a risk for human disease, zoonotic visceral leishmaniasis (ZVL) [3,5]. In the Mediterranean region, the annual incidence of ZVL is estimated to range from 1,200 up to 2,000 [8].

CanL endemicity is associated with the distribution and abundancy of its vectors, phlebotomine sand flies. In southern Europe, 5 species are proven vectors of CanL [9], of which *Phlebotomus perniciosus* is the most important. During the bite, the sand fly injects saliva containing a cocktail of bio-active molecules with anti-hemostatic, anti-inflammatory and immune-modulatory activities into the host skin (reviewed in [10]). These molecules facilitate the blood-feeding process of the sand fly and trigger a humoral immune response in the host. It is well-known that the amount of host anti-saliva IgG antibodies (Abs) correlates with the level of exposure to sand flies [11,12]. Furthermore, previous studies showed a clear fluctuation of the Ab response during longitudinal sampling of dogs over two transmission seasons [26], suggesting that proteins present in sand fly saliva can be a useful tool to evaluate the efficacy of vector control programs. For example, previous studies on mosquitoes [13–15] and triatomine bugs [16] have shown that a reduction in vector density observed after the implementation of insecticide treated nets (ITNs) correlates with a reduction in anti-vector salivary Ab-response. With regard to sand flies, only one study performed in India and Nepal measured anti-*P*. *argentipes* Abs to evaluate the use of ITNs [17]. Performing large-scale serological studies to detect host exposure to sand fly bites was limited in the past due to the fact that dissecting large amounts of sand fly salivary glands is a demanding and labour-intensive process. Besides, the use of whole SGH is subject to protein content variability, dependent on the age of the sand fly at the time of dissection [18] and might antigenically cross-react with taxonomically closely related sand fly species (reviewed in [19]). Therefore, using specific antigenic recombinant sand fly salivary proteins as a replacement to the use of whole SGH has gained more attention [20–23].

Previously the specific antibody response (IgG) against the salivary protein SP03B from *P*. *perniciosus* was proposed as a valid exposure marker across regions endemic for CanL [22]. This study demonstrated the presence of similar antigenic epitopes in the recombinant SP03B protein compared to its native form, and indicated a substantial antigenic cross-reactivity amongst *P*. *perniciosus* populations from Campania, Umbria and the metropolitan Lisbon region [22]. The SP03B salivary protein belongs to the family of yellow-related proteins [24] and was previously shown to possess binding activity for pro-haemostatic and pro-inflammatory biogenic amines in *Lutzomyia longipalpis* [25]. Recently, recombinant yellow-related proteins were subject of epidemiological studies to determine the levels of specific anti-vector salivary Abs in naturally bitten hosts [20,21,26,27]. All of these studies used indirect enzyme-linked immunosorbent assays (ELISA). Although this approach is useful in laboratory settings, a practical tool that can immediately be used in the field and that allows a fast screening of hosts living in endemic areas is called for.

In this study, we evaluated our newly proposed colloidal gold immunochromatographic test (ICT)–the rSP03B sero-strip—against the standard ELISA method. The use of colloidal gold ICTs has first been described by Osikowicz and Beggs for the qualitative detection of human chorionic gonadotropin (hCG) [28] and since then deployed in a broad range of fields. However, with regard to the detection of IgG Abs against arthropod saliva, no such test has been described yet. Therefore, we propose the first colloidal gold ICT in the field of vector exposure. Since our test is very straightforward—no special equipment or skill is required—it can be easily operated in non-laboratory settings to rapidly screen large cohorts of dogs for exposure to *P*. *perniciosus*.

## Methods

### Ethics statement

Reuse of canine sera samples obtained during previous studies [11,26] was approved by the Ethical Board of Charles University. Ethical approval of sera samples collected from non-bitten laboratory-bred Beagles housed at the University of Zaragoza, Spain (UNIZAR) was obtained during another ongoing study, protocol PI44/17. All sampling complied with the European guidelines on the protection of animals (Directive 2010/ 63/UE).

### Sources of sera

Forty-two sera samples from laboratory-bred Beagles experimentally exposed to *P*. *perniciosus* were used to prepare the first prototype of the test. These dogs were individually exposed to approximately 200 *P*. *perniciosus* females during a previous study. The sampling protocol is described in more detail in [11]. Negative control sera were collected from 29 non-bitten laboratory-bred Beagles housed at the University of Zaragoza, Spain (UNIZAR). Furthermore, sera samples from 24 laboratory-bred Beagles born in a breeding facility located in northern France were used as negative controls.

### Antigens

The antigen (Ag) used for the preparation of the rSP03B sero-strip is a bacterially expressed 43kDa yellow-related recombinant protein of *P*. *perniciosus* (rSP03B, Genbank accn. DQ150622). The recombinant protein was obtained from Apronex s.r.o. (Prague) as described in [29] and was expressed with the *Escherichia coli* BL21 (DE3) expression system in the pET28b vector (Novagen) with a poly-His tag (6 histidines). The protein was isolated under denaturing conditions with 8M urea (50mM Tris, pH 8, 300mM sodium chloride) and prepared for usage in the rSP03B sero-strip by gradually dialyzing it to a final concentration of 0M urea (PBS 1x, pH 6) using Slide-A-Lyzer mini dialysis units (10K MWCO, 0.1mL), following the manufacturer’s protocol. The UV absorbance value of the protein was determined by Nanodrop at 280nm. The protein concentration was then quantified by means of the known molar extinction coefficient of the protein.

The Ags used for the ELISA include the rSP03B salivary protein and the whole salivary gland homogenate (SGH) from *P*. *perniciosus*. A colony of *P*. *perniciosus* was reared under standard conditions as described in [30] and salivary glands were dissected from 4–6 days-old female sand flies, pooled in 20mM Tris buffer with 150mM NaCl and stored at -20°C. Before use, the SGH was prepared by disrupting the salivary glands during 3 freeze-and-thaw cycles in liquid nitrogen.

### Indirect enzyme-linked immunosorbent assay (ELISA)

All sera samples were analyzed by an indirect enzyme-linked immunosorbent assay (ELISA) that measures anti-*P*. *perniciosus* IgG. The ELISA was performed in accordance with previous studies [26], with minor modifications. Briefly, flat bottom microtiter plates (Immulon) were coated with *P*. *perniciosus* salivary gland homogenate (SGH) (0.2 salivary gland per well) or with rSP03B (5μg/ml) in 20mM carbonate-bicarbonate buffer (pH 9, 100μl/well) and incubated overnight at 4°C. The plates were washed with PBS + 0.05% Tween 20 (PBS-Tw) and blocked with 6% (w/v) low fat dry milk diluted in PBS-Tw. Canine sera diluted in 2% (w/v) low fat dry milk/PBS-Tw was added to the wells (100μl/well). Sera were diluted at 1/200 and 1/100 for SGH and rSP03B, respectively. After 90min incubation at 37°C, the plates were incubated at 37°C for 45min with secondary Abs (polyclonal anti-dog IgG-horseradish peroxidase (HRP), Bethyl laboratories, 100μl/well) diluted 1:9000 in PBS-Tw. The ELISA was developed using an orthophenylendiamine (OPD) solution in a phosphate-citrate buffer (pH 5.5) with 0.1% hydrogen peroxide. The reaction was stopped after 5min with 10% sulfuric acid and absorbance (OD value) was measured at 492nm using a Tecan Infinite M200 microplate reader (Schoeller). Each serum was tested in duplicate.

### Preparation of the rSP03B sero-strip

**Fig 1:**
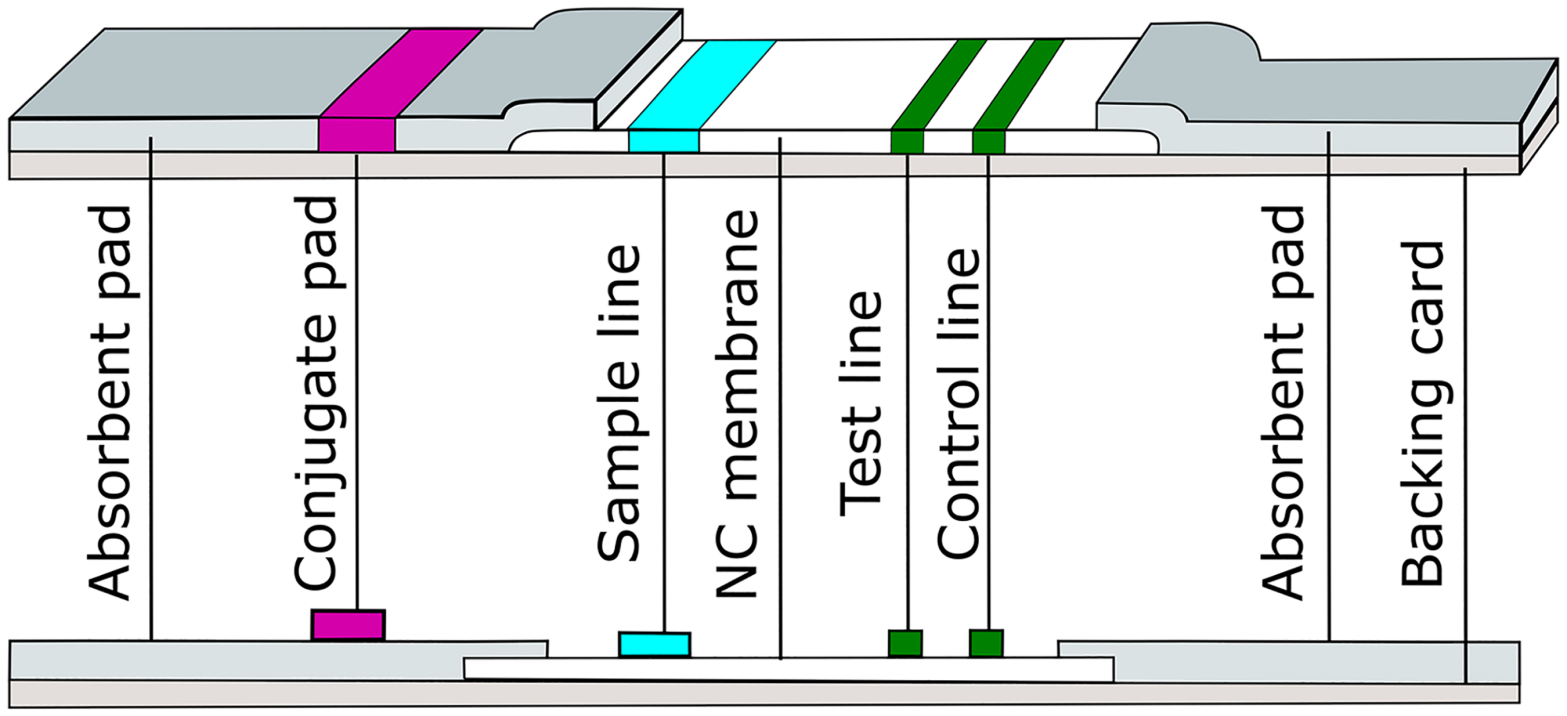
Composition of the rSP03B sero-strip. The rSP03B sero-strip consists of a backing card to which a lower absorbent and an upper absorbent pad are attached. Both of these pads overlap an NC membrane located in the middle of the test. On the NC membrane 3 lines are coated: the sample line, a test line and a control line. The lower absorbent pad is impregnated with a colloidal gold-conjugate (conjugate pad), consisting of a mixture of the conjugates for the test and the control line. NC = nitrocellulose.

The rSP03B sero-strip is composed of a lower absorbent pad and an upper absorbent pad that both overlap a nitrocellulose (NC) membrane located in the middle of the test (Fig 1). The lower absorbent pad is impregnated with a colloidal gold-conjugate consisting of a mixture of one conjugate for the test line and one for the control line. The conjugate for the test line was prepared by coupling a polyclonal anti-dog IgG Ab (Bethyl laboratories) to colloidal gold nanoparticles. Secondly, the control conjugate was prepared by coupling a chicken Ab from non-immunized chickens to colloidal gold nanoparticles. The coupling of both conjugates was followed by a saturation step (gold blocking buffer, Coris BioConcept). On the NC membrane 3 lines were coated. The first line consists of sample deposition line and enables a complete migration of the sample. The second line is the test line on which the dialyzed rSP03B protein is coated (0.6mg/ml, 0.1μl/mm) and the third line (migration control) consists of a goat anti-chicken Ab (GAC) that binds to the colloidal gold ‘control’ conjugate.

### Principle of the rSP03B sero-strip

**Fig 2:**
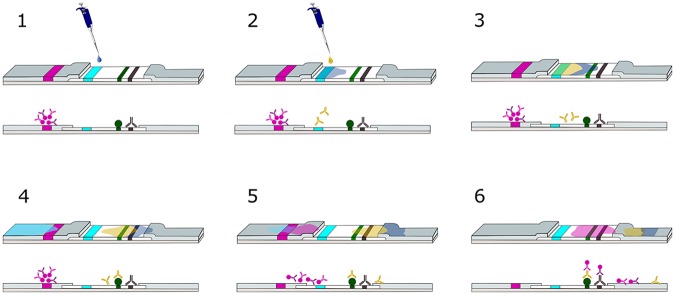
Stepwise description of the rSP03B sero-strip principle. To start the test a buffer is deposited on the sample line (NC membrane) (1). Immediately after applying the buffer, the sample is spotted on the same position (2). Both the buffer and the sample will migrate to the upper part of the strip. The anti-rSP03B Abs present in the sample get captured by the rSP03B Ag coated on the test line (3). The strip is then immediately dipped into the migration buffer solution (4). After which the colloidal gold conjugate mixture gets hydrated and starts migrating upwards together with the moving liquid (5). The anti-dog IgG Ab-gold conjugate binds to the dog IgG Abs on the test line. The colloidal gold control conjugate is captured by the GAC Abs present at the control line (6). NC, Nitrocellulose; Abs, Antibodies; Ag, Antigen; GAC, Goat anti-chicken.

**Fig 3:**
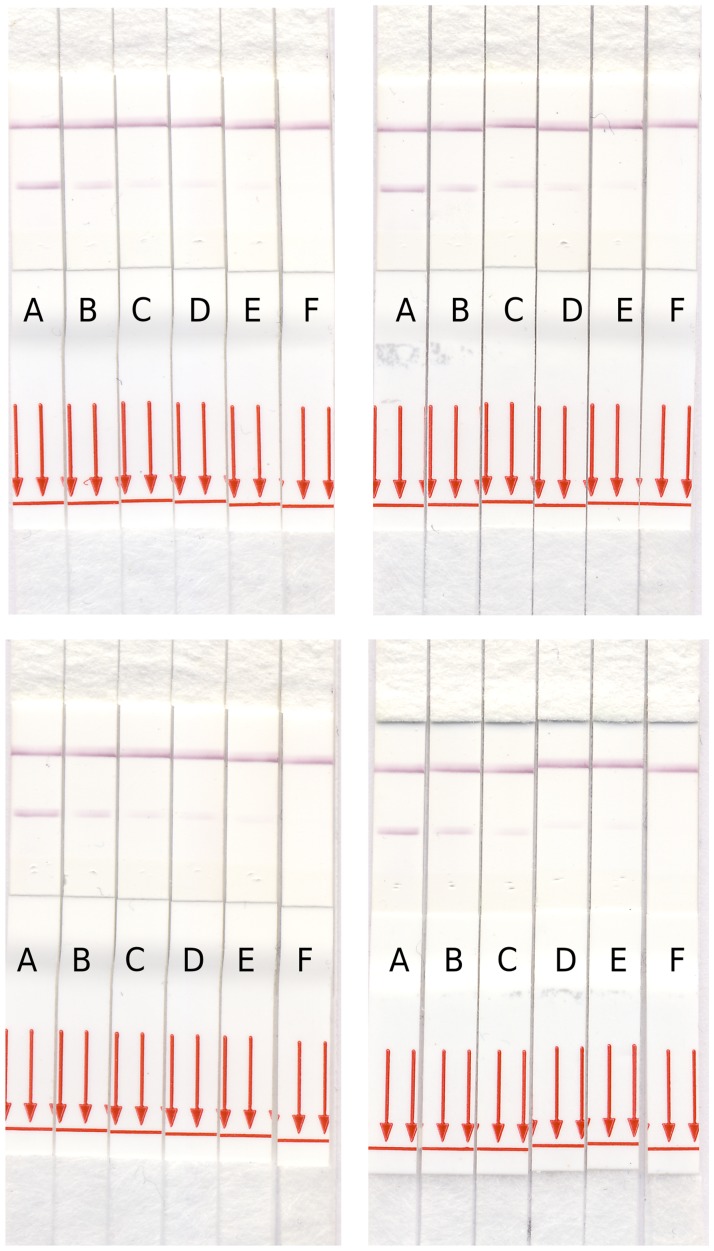
Intensity of test line relates to amount of target Ab deposited. Dilution series of four highly positive sera samples indicate that the intensity of the purple color at the test line decreases when a lower amount of target Ab is deposited on the strip. The sample appears negative when a dilution of 1/50 is used. A: undiluted sample; B: 1/10 sample dilution; C: 1/20 sample dilution; D: 1/30 sample dilution; E: 1/40 sample dilution; F: 1/50 sample dilution.

**Fig 4:**
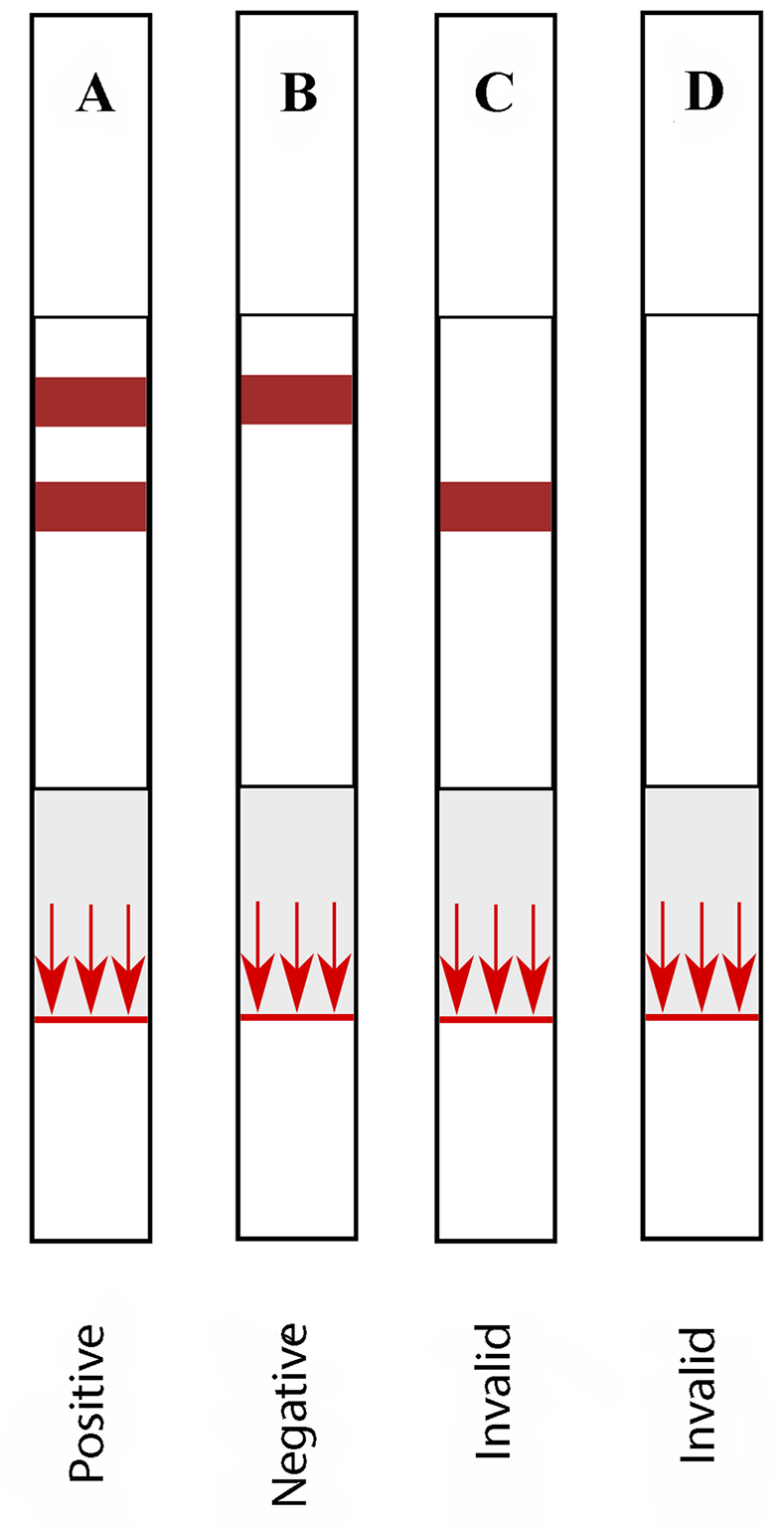
Interpretation of the results. The test is only valid if the migration control line is present (A and B). A positive test result is observed when two lines (test and control) are visible on the NC membrane (A). The test is considered negative when only the control line is present (B). When the migration control line is not present, the test is considered invalid and should be redone (C and D). NC, Nitrocellulose.

In order to launch the test, a buffer (3μl; HC dilution buffer, Coris BioConcept) is applied to the sample line (blue) on the NC membrane (step 1 in Fig 2). Immediately after applying the buffer, the serum sample (1μl) is deposited on the same spot (step 2 in Fig 2). This will cause the sample to start migrating to the upper part of the strip, where the anti-rSP03B Abs present in the serum sample will be captured by the rSP03B Ag coated on the test line (step 3 and 4 in Fig 2). Directly after deposition of the sample, the strip is dipped into the migration buffer solution (step 4 in Fig 2; Ly-B dilution buffer, Coris BioConcept). The colloidal gold conjugate, consisting of the test- and control-conjugate mixture, gets hydrated and starts migrating upwards together with the moving liquid (step 5 in Fig 2). Once arrived at the test line, the conjugate will recognize dog IgG Abs bound to the rSP03B Ag coated at the test line (step 6 in Fig 2). While moving further up the NC membrane, the GAC Abs present at the control line will capture the colloidal gold ‘control’ conjugate, resulting in the appearance of a purple color. This is an essential part of the rSP03B sero-strip as it ensures that the migration went well and the strip is functioning properly. The test is run for 15min and excess buffer solution is absorbed by the upper absorption pad. Intensity of the purple color at the test line relates to the amount of target Ab present in the sample and is visually inspected (Fig 3). The test is only valid if the migration control line is visible. A positive test result is observed when two lines (test and control) are visible on the NC membrane. The test is considered negative when only the control line is present (Fig 4).

### Statistical analyses

All samples tested in ELISA were tested in duplicate. The coefficient of variation (CoV) was calculated for each duplicate sample. If the CoV was more than 15% the sample was retested. Since plates were ran on multiple days, positive control sera (PC, n = 2) and negative control sera (NC, n = 2) were included in each plate. In order to correct for interplate variability, the obtained OD values were standardized (SOD) according to the following formula:

$$SOD\;(\%)=\frac{OD\;sample}{Average\;OD\;PC}\;x\;100$$

ELISA cut-offs were determined by the mean of the OD values plus 3 standard deviations (SD) obtained from non-bitten negative controls. Graphical representation of the distribution of the SOD (%) was visualized using the “beeswarm” package in R software [31,32]. The results of the rSP03B sero-strip were classified according to the intensity of the observed band, starting from (0) a negative test result; (1) a very faint signal; (2) a low positive signal; (3) a positive but less intense signal than the control band and (4) a strong positive signal, same intensity as the control band. All samples classified in categories (1), (2), (3) and (4) were considered positive. The data was graphically represented using the “ggplot2” package in R software [31,33]. The degree of agreement between SGH-ELISA, rSP03B-ELISA and the rSP03B sero-strip was measured by Cohen’s Kappa according to the methods of Jacob Cohen [34]. Furthermore, the percentage of agreement between different serological methods was measured and the Mc Nemar’s χ^2^ test in R software [31] was used to test for significant differences in agreement between the golden standard SGH-ELISA, the rSP03B-ELISA and the rSP03B sero-strip. Correlation between both ELISA tests was analyzed using Pearson’s r correlation coefficient and graphically visualized using the “ggplot2” package in R software [31,33].

## Results

### Sensitivity and specificity of each serological method

**Fig 5:**
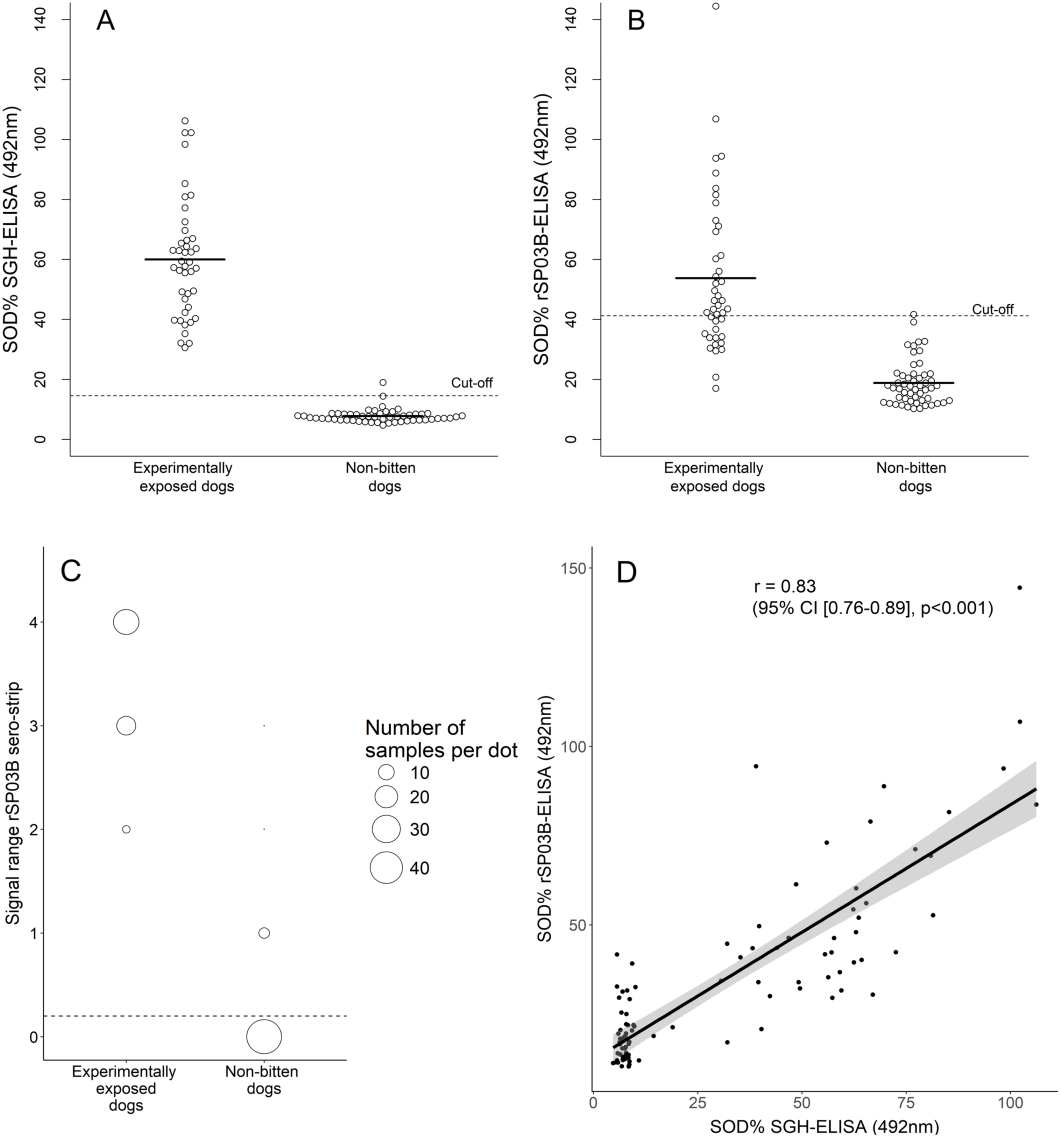
Distribution of results from ELISA and the rSP03B sero-strip. SOD (%) distribution of positive sera samples (experimentally exposed dogs) and negative sera samples (non-bitten dogs) tested by SGH-ELISA (A) and rSP03B-ELISA (B) is shown. The cut-off for each ELISA was calculated by the mean of non-bitten negative control sera +3SD. Results from the rSP03B sero-strip were classified according to the intensity of the observed band, starting from (0) a negative test result; (1) a very faint signal; (2) a low positive signal; (3) a positive but less intense signal than the control band and (4) a strong positive signal, same intensity as the control band. All samples classified in categories (1), (2), (3) and (4) were considered positive. Distribution of positive sera samples and negative sera samples is shown in (C). Correlation between SOD (%) values of SGH-ELISA and rSP03B-ELISA was performed using Pearson’s r correlation and is shown in (D). SD, Standard Deviation; SOD, Standardized Optical Density; SGH, Salivary Gland Homogenate; r, Correlation index; CI, Confidence Interval.

The specificity and sensitivity of the 3 serological methods used were calculated based on the results from experimentally exposed dogs and negative control sera (Table 1). The distribution of the results in SOD (%) is shown in Fig 5. Results from the SGH-ELISA and rSP03B-ELISA were first classified as being positive or negative according to their respective cut-off value (Fig 5A and 5B). All 42 sera of dogs experimentally exposed to *P*. *perniciosus* appeared positive on the SGH-ELISA. However, only 29 out of these 42 samples were classified as being positive in the rSP03B-ELISA. Additionally, a single serum sample out of 53 negative control sera was found to be positive in the SGH-ELISA, and rSP03B-ELISA. These observations resulted in a sensitivity of 100% and 69% for the SGH-ELISA and rSP03B-ELISA, respectively, and a specificity of 98.11% for both methods. Results obtained from our newly prepared rSP03B sero-strip were classified according to the intensity of the observed band. The distribution of the samples tested by the rSP03B sero-strip is shown in Fig 5C. All 42 sera samples of experimentally exposed dogs presented 2 purple bands—one on the test line and one on the control line—resulting in a sensitivity of 100%. On the other hand, 7 out of 53 sera from non-bitten negative controls were also found positive with our rSP03B sero-strip, giving it a specificity of 86.79%. Importantly, raising the detection limit of the rSP03B sero-strip until category (1) will be considered negative results in a specificity of 96.23%, without changing the sensitivity.

**Table 1:** Sensitivity and specificity of each serological method (SGH-ELISA, rSP03B-ELISA and the rSP03B sero-strip).

	True positives	True negatives	Sensitivity	Specificity
**SGH-ELISA**	42/42	52/53	100% (95% CI [89.56%–100%])	98.11% (95% CI [88.62%–99.90%])
**rSP03B-ELISA**	29/42	52/53	69% (95% CI [52.76%–81.89%)	98.11% (95% CI [88.62%–99.90%])
**rSP03B sero-strip**	42/42	46/53	100% (95% CI [89.56%–100%])	86.79% (95% CI [74.05%–94.09%])

The predictive values for each method were calculated based on results from experimentally exposed dogs and negative control sera. CI, Confidence Interval.

### Comparison of rSP03B-ELISA and rSP03B sero-strip with the SGH-ELISA

Since the SGH-ELISA showed the highest sensitivity and specificity amongst all 3 methods, it was set as the golden standard against which the rSP03B-ELISA and the rSP03B sero-strip were evaluated (Table 2). The sensitivity and specificity of the rSP03B sero-strip as compared to the SGH-ELISA were 97.67% and 87%, respectively. A percentage of agreement of 91.58% was obtained with no systematic difference between the proportions of positive responses from these two methods (McNemar χ^2^ P > 0.05). Furthermore, the Cohen’s kappa value between the rSP03B sero-strip and the SGH-ELISA was 0.83, suggesting an almost perfect strength of agreement between these two methods. With regard to the rSP03B-ELISA a sensitivity and specificity of 67.44% and 98% were measured, respectively. The percentage of agreement between the rSP03B-ELISA and the SGH-ELISA was set at 84.21% with a Cohen’s kappa value of 0.67, suggesting a substantial strength of agreement. However, McNemar χ^2^ tested a significant systematic difference between the proportions of positive responses from the rSP03B-ELISA and the SGH-ELISA (P < 0.05). Interestingly, when raw OD-values of both ELISA methods were compared, a correlation of 83.43% was obtained (95% CI [76.06%– 88.67%], P < 0.001) (Fig 5D).

**Table 2:** Comparison of rSP03B-ELISA and rSP03B sero-strip with standard SGH-ELISA.

		SGH-ELISA		Total	Sensitivity	95% CI	Specificity	95% CI	Percent agreement (%)	Mc Nemar χ^2^	Kappa
		+	-								
**rSP03B-ELISA**	**+**	29	1	95	67.44%	51.34%–80.46%	98%	88.42%–99.90%	84.21	*P* < .05 (0.0019)	0.67
	**-**	14	51								
**rSP03B sero-strip**	**+**	42	7	95	97.67%	86.20%–99.88%	87%	73.60%–93.97%	91.58	*P* > .05 (0.0771)	0.83
	**-**	1	45								

Comparison between the previously described rSP03B-ELISA and the golden standard SGH-ELISA is shown in the 1st row. Comparison between the newly proposed rSP03B sero-strip and the golden standard SGH-ELISA is shown in the 2nd row. (+), positive result; (-), negative result; CI, Confidence Interval.

## Discussion

Previous studies addressing the level of host exposure to sand fly bites consistently used ELISA methods to determine the levels of specific anti-vector salivary Abs. Although this method is useful in laboratory settings, a rapid test that can aid in vector control by allowing a consistent screening of hosts in the field has not yet been described. Here, we developed a new rapid test that can be immediately used in the field to screen dogs living in endemic CanL areas for the presence of anti-*P*. *perniciosus* IgG Abs. In the proposed rSP03B sero-strip, the yellow-related rSP03B *P*. *perniciosus* salivary protein was used as Ag, previously proposed as a valid exposure marker for *P*. *perniciosus* across its entire area of distribution [22]. The principle of the rSP03B sero-strip is similar to an indirect ELISA; first specific canine Abs present in the sample bind to the *P*. *perniciosus* salivary Ag immobilized on the NC membrane of the sero-strip, after which the interaction is visualized by an anti-dog IgG Ab gold-conjugate.

In this study, 3 serological methods to define the level of *P*. *perniciosus* salivary IgG Abs were compared: the golden standard SGH-ELISA, the previously described rSP03B-ELISA [22] and our newly proposed rSP03B sero-strip. The results highlight the SGH-ELISA as being the most sensitive (100%) and most specific (98.11%) amongst all 3 methods. When comparing the performance values of the rSP03B-ELISA and the rSP03B sero-strip, the rSP03B sero-strip appears to have the highest sensitivity (69% vs. 100%, respectively) whereas the rSP03B-ELISA shows the highest specificity (98.11% vs. 86.79%, respectively). Finally, the rSP03B sero-strip was shown to have an almost perfect agreement with the SGH-ELISA without any significant differences, overall suggesting that the sero-strip performs better than the rSP03B-ELISA. Moreover, the performance of the sero-strip can be further improved as was shown by an increased specificity (up to 96%) without a loss in sensitivity when very faint results would be considered negative. This can be achieved by the use of a strip reader which will classify the results according to a pre-defined cut-off value for the sero-strip.

Although our results indicate that our newly proposed rSP03B sero-strip is a valid replacement for the rSP03B-ELISA, the performance values of the rSP03B-ELISA should be taken with caution. When raw OD-values of both ELISA methods were compared, a correlation of 83.43% was achieved. This high correlation between the rSP03B-ELISA and the SGH-ELISA suggests that classifying the samples according to positivity might be the reason for the high number of false negatives in the rSP03B-ELISA. The higher cut-off value observed in the rSP03B-ELISA could be explained by a non-specific reaction that takes place in the ELISA between the negative control samples and bacterial proteins that possibly co-purified with the rSP03B protein [35] and might be overcome by producing the protein in a different expression system (e.g. mammalian cells). The reason why these false negative results do not occur to the same extent with the rSP03B sero-strip is explained by the fact that this is a qualitative method and is therefore not dependent on a specific cut-off value. Thus, any observed signal will be classified as positive.

In summary, we developed a simple and rapid colloidal gold ICT based on the bacterially expressed recombinant protein rSP03B that is able to replace the ELISA method used in numerous previous studies [11,20–22,26]. Our rSP03B sero-strip showed to be highly sensitive (100%) and specific (86.79%) in the detection of IgG Abs against *P*. *perniciosus* saliva. The test is easily operated with no requirements for skilled personnel or specialized equipment. However, in order to confirm the field detection accuracy and applicability of the test, further evaluation of canine populations exposed to various frequencies of sand fly bites and validation of the test with whole canine blood is required. Additionally, it is worth to mention that potential cross-reactivities between Abs recognizing salivary proteins of closely related sand fly species has been observed in previous studies [36]. Therefore, a cross-reaction between the rSP03B protein and Abs against salivary proteins of closely related sand fly species of subgenus *Larrousius* is likely to occur, hence rendering the sero-strip useful for estimating exposure to other vectors of CanL in the Mediterranean basin. However, due to the lack of colonies of sand fly species co-occurring with *P*. *perniciosus* [37] this cross-reaction cannot be tested at the moment. Unfavorable cross-reactions with other haematophagous insects are very unlikely but we suggest to reflect on this during further studies.
